# ^123^I‐BMIPP single‐photon emission computed tomography for diagnosing chest pain in patients with nonobstructive coronary angiograms: Takotsubo or vasospasm?

**DOI:** 10.1002/ccr3.1134

**Published:** 2017-08-15

**Authors:** Yuta Seko, Kenichi Sasaki, Takao Kato, Moriaki Inoko

**Affiliations:** ^1^ Cardiovascular Center Tazuke Kofukai Medical Research Institute Kitano Hospital Osaka Japan; ^2^ Department of Cardiovascular Medicine Kyoto University Graduate School of Medicine Kyoto Japan

**Keywords:** Coronary vasospasm, ischemic memory, Takotsubo cardiomyopathy

## Abstract

The decreased 123I‐BMIPP uptake, while having normal perfusion, in the correspondent coronary artery territory is caused by a delayed metabolic recovery after the recovery of perfusion. This phenomenon termed ischemic memory can clearly differentiate vasospastic angina from other chest pain syndromes.

## Question

What is the differential diagnosis and the next diagnostic step of the patient with recurrent angina at rest without obvious obstruction of coronary artery?

## Answer

Takotsubo cardiomyopathy and vasospastic angina are the differential diagnoses. The decreased uptake of a fatty acid tracer in the correspondent coronary artery territory suggested vasospastic angina, which was confirmed by ergonovine provocation.

**Figure 1:**
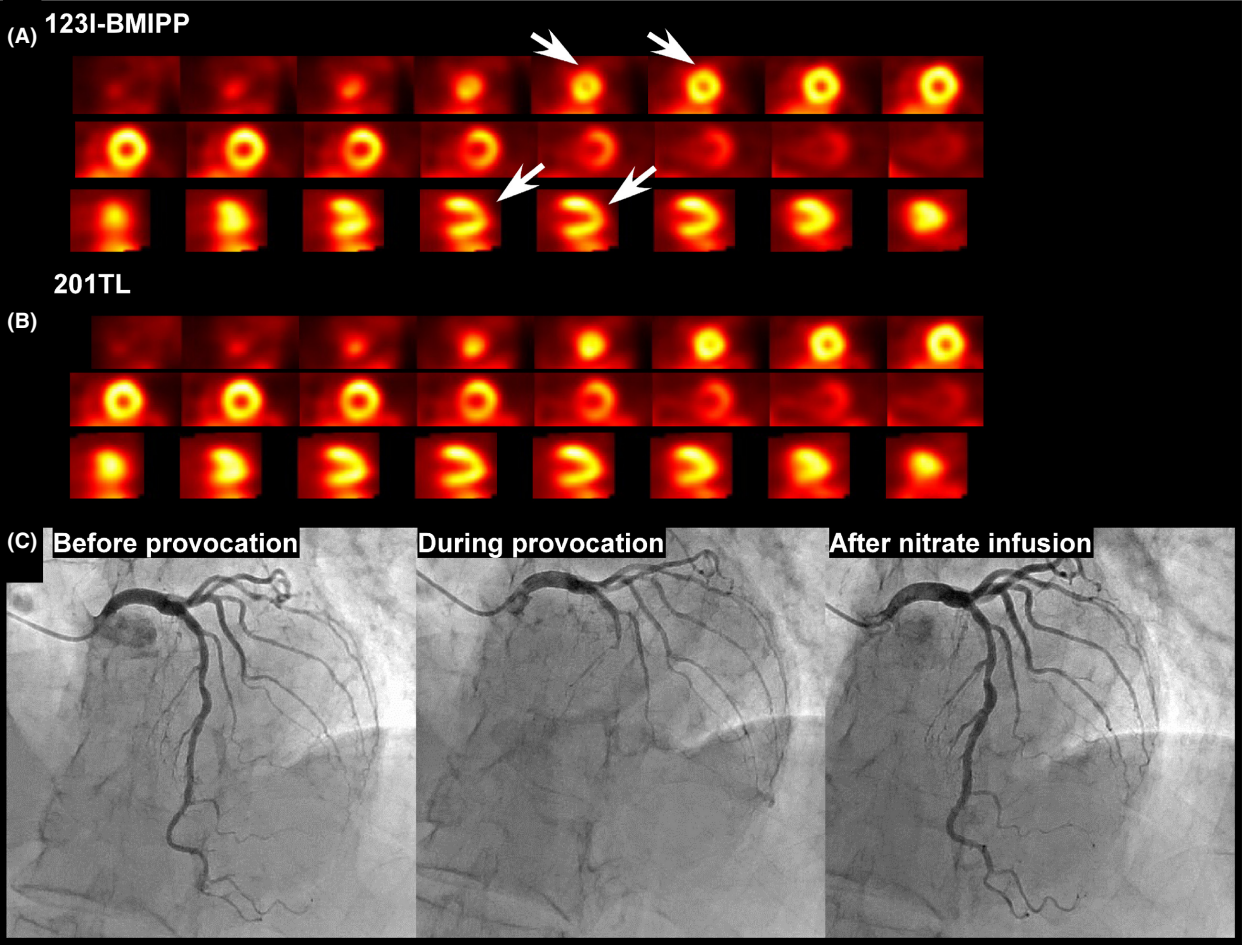
(A) ^123^I‐bmethyl‐p‐iodophenylpentadecanoic acid (BMIPP) images. The decreased uptake of ^123^I‐BMIPP, a fatty acid tracer, was observed according to the coronary artery territory of the left anterior descending artery (LAD). The basal septal portion was spared on the BMIPP images, which means that the ischemia was provoked after the first septal branch of the left anterior descending artery (LAD). (B) ^201^Thallium images showed normal perfusion. (C) Ergonovine provocation induced spasm and a subsequent obstruction of the LAD after the first septal branch.

A 69‐year‐old woman who was admitted for colon cancer treatment complained of sudden‐onset chest pain that lasted for 20 min, with ST elevations in leads V3 and V4 on ECG and akinesis of the apex on echocardiography. Elevated troponin I levels were observed with a peak value of 0.493 ng/mL without elevated creatine kinase levels. No obstruction was noted on the emergent angiography, while left ventriculography revealed apical ballooning, which indicated Takotsubo cardiomyopathy or coronary spasm as the etiology. Repeated chest pain with similar ST elevation at rest was observed on days 2 and 3, even after the oral administration of a calcium channel blocker. On day 5, decreased uptake of ^123^I‐bmethyl‐*p*‐iodophenylpentadecanoic acid (BMIPP) was observed at the apical anterior wall with spared basal septum (Fig. 1A) despite normal perfusion of ^201^Thallium (Fig. 1B). This suggested coronary spasm of the distal portion after the first septal branch of the left anterior descending artery (LAD). Ergonovine provocation induced spasm and a subsequent obstruction of the LAD after the first septal branch (Fig. 1C). Whether an ^123^I‐BMIPP abnormality is in accordance with the corresponding coronary territory is key to distinguishing between vasospasm and Takotsubo cardiomyopathy 1, 2.

## Authorship

YS and TK: drafted the article. YS, KS, TK, and MI: revised the article, gave the final approval of the article, and have accountability for all aspects of the work.

## Conflict of Interest

None declared.
